# Upregulation of KSRP by miR-27b provides IFN-γ-induced post-transcriptional regulation of CX3CL1 in liver epithelial cells

**DOI:** 10.1038/srep17590

**Published:** 2015-12-03

**Authors:** Zijie Xia, Yajing Lu, Xiaoqing Li, Tiebo Mao, Xian-Ming Chen, Rui Zhou

**Affiliations:** 1 School of Basic Medical Sciences, Wuhan University, Hubei 430071, China; 2 Institute of Hematology, Union Hospital, Tongji Medical School, Huazhong University of Science and Technology, Wuhan, Hubei 430000, China; 3 Department of Medical Microbiology and Immunology, Creighton University School of Medicine, Omaha, NE 68178, USA

## Abstract

Aberrant cellular responses to pro-inflammatory cytokines, such as IFN-γ, are pathogenic features in many chronic inflammatory diseases. A variety of feedback regulatory pathways have evolved to prevent an inappropriate cellular reaction to these pro-inflammatory cytokines. CX3CL1 is a unique chemokine and plays an important role in chronic liver diseases. We report here that IFN-γ stimulation induces a transient CX3CL1 production in liver epithelial cells (i.e., hepatocytes and biliary epithelial cells). This transient CX3CL1 production is accompanied with a destabilization of CX3CL1 mRNA associated with the induction of the KH-type splicing regulatory protein (KSRP). IFN-γ treatment of liver epithelial cells decreases expression level of miR-27b, a miRNA that targets the 3′ untranslated region of KSRP mRNA resulting in translational suppression. Induction of KSRP following IFN-γ stimulation depends on the downregulation of miR-27b. Functional manipulation of KSRP or miR-27b caused reciprocal alterations in CX3CL1 mRNA stability in liver epithelial cells. Moreover, transfection of miR-27b precursor influences CX3CL1-associated chemotaxis effects of biliary epithelial cells to Jurkat T cells. These findings suggest that miR-27b-mediated post-transcriptional suppression controls the expression of KSRP in liver epithelial cells, and upregulation of KSRP destabilizes CX3CL1 mRNA, providing fine-tuning of cellular inflammatory reactions in response to IFN-γ stimulation.

The inflammatory response is a double-edged sword, as excessive inflammation itself can exacerbate tissue damage^1^,^2^. Chronic inflammation and cellular injury are common pathogenic features for a variety of important hepatobiliary diseases, such as chronic type C hepatitis^3^. Persistent inflammation in the liver of patients with these diseases is usually accompanied with increased expression of multiple inflammatory mediators, including inflammatory cytokines/chemokines^4^. To limit the undesirable consequences of excessive inflammation, liver epithelial cells (i.e., hepatocytes and biliary epithelial cells) have developed regulatory strategies to control the initiation and resolution of inflammatory response^5^,^6^. The coordinated expression of various components of cellular inflammatory response involves multiple steps that determine rates of gene transcription, translation, and mRNA decay^6^,^7^. Although transcription is an essential first step in the regulation of gene expression, post-transcriptional regulation of translation and mRNA decay is key to control protein synthesis from transcribed mRNAs^6^. It is now apparent that 3′-untranslated region (3′UTR)-mediated RNA stability and translational activation play an important regulatory role in the post-transcriptional regulation of protein synthesis^7^,^8^. Nevertheless, the role for 3′UTR-mediated post-transcriptional regulation in the coordination of liver epithelial cell inflammatory responses remains to be defined.

Several RNA-binding proteins, including the KH-type splicing regulatory protein (KSRP, also known as KHSRP), tristetraprolin (TTP) and Hu antigen R (HuR), recognize AU-rich elements (AREs) within the 3′UTRs of mRNAs and control their half-life time in the cytoplasm^7^,^8^,^9^. In this regard, KSRP interacts with these mRNAs that have the AREs within their 3′UTRs and is a key mediator of mRNA decay^10^. Some KSRP-regulated mRNAs code proteins are key to cellular inflammatory response, including mRNAs for inducible NO synthase (iNOS) and cyclooxygenase-2 (COX-2)^11^. The 3′UTR is also critical to miRNA-mediated post-transcriptional gene regulation. In mammalian cells, miRNAs identify targets based on complementarity between each miRNA and 3′UTR of target mRNAs, resulting in mRNA cleavage and/or translational suppression^12^.

The chemokine CX3CL1 (also known as fractalkine) is a unique member of the CX3C family; and it binds only to the unique ligand of its receptor, CX3CR1^13^. Unlike other chemokines, CX3CL1 is expressed as a membrane-bound form (95–100 kDa) and can also be shed in a soluble chemotactic form (60–80 kDa)^13^,^14^. Membrane-bound CX3CL1 is known to function as an adhesion molecule to interact with immune cells that express CX3CR1, including CD4 + and CD8 + T-cells, NK cells, and monocytes^15^. Recent evidence shows that increased level of CX3CL1 in the liver is associated with severe inflammatory liver diseases^16^. In our previous studies, we demonstrated that induction of CX3CL1 expression in biliary epithelial cells upon microbial challenge involves downregulation of miR-424 and miR-503^17^. Histone deacetylases and NF-ĸB signaling coordinate downregulation of the *mir-424-503* gene and promote biliary mucosal defense through CX3CL1 induction in biliary epithelial cells^17^. Using *in vitro* and *in vivo* models of biliary Cryptosporidiosis, we found that KSRP is a target of miR-27b in biliary epithelial cells; and post-transcriptional suppression of KSRP by miR-27b stabilizes iNOS mRNA and facilitates TLR4-mediated biliary epithelial defense against *Cryptosporidium parvum* infection^18^.

IFN-γ, a type II interferon with important immunomodulatory properties, plays a critical role in mediating liver inflammatory responses. While IFN-γ is key to innate and adaptive immunity against viral and intracellular bacterial infection in the liver, uncontrolled IFN-γ signaling may be pathogenic, contributing to the pathogenesis of chronic autoimmune and inflammatory hepatobiliary diseases^19^. IFN-γ triggers transactivation of many inflammatory genes and increases their expression in liver cells^19^,^20^. In this report, we investigated the expression of KSRP in liver epithelial cells following IFN-γ stimulation, its relationship to miR-27b-mediated translational suppression, and finally, its impact on cellular inflammatory responses in the liver. Our findings implicate that relief of miR-27b-mediated post-transcriptional suppression of KSRP destabilizes CX3CL1 mRNA in liver epithelial cells, a process that may provide fine-tuning of liver epithelial inflammatory reactions to IFN-γ.

## Results

### IFN-γ stimulation induces a transient CX3CL1 expression

**Figure 1:**
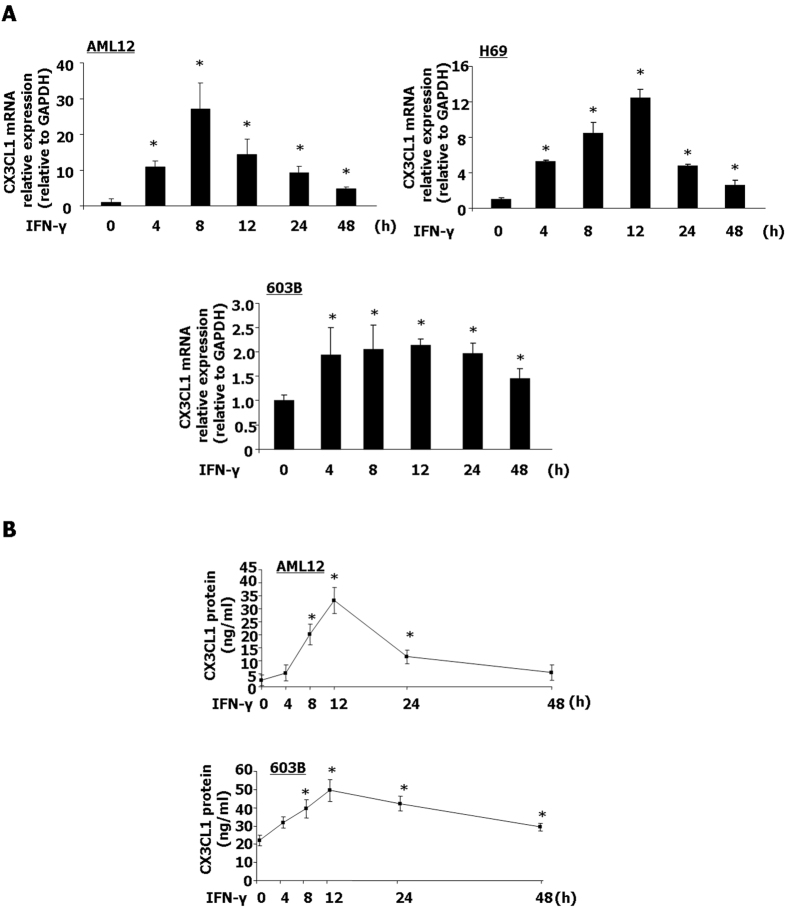
IFN-γ stimulation induces a transient CX3CL1 expression. (**A**) AML12; H69 and 603B cells were exposed to IFN-γ (10 ng/ml) for up to 48 h, followed by real-time PCR analysis for CX3CL1 mRNA. (**B**) AML12 and 603B cells were stimulated by IFN-γ (10 ng/ml) for up to 48 h, CX3CL1 protein in cell lyses (black square) was measured by ELISA. Quantification of CX3CL1 mRNA levels and protein levels from three independent experiments was shown. *p < 0.05 vs. non-treated cells (in **A,B**).

We first assessed expression of CX3CL1 at the mRNA level and protein level in cultured hepatocytes (AML12) and biliary epithelial cells (H69 and 603B) in response to IFN-γ stimulation. An increased CX3CL1 mRNA level was detected in AML12 cells following IFN-γ treatment for 8 h (Fig. 1A). Interestingly, CX3CL1 mRNA level started to decline at 8 h and continued to decrease till 48 h following IFN-γ stimulation (Fig. 1A). In H69 and 603B cells, levels of CX3CL1 mRNA started to increase at 4 h and began to decline after 12 h following IFN-γ treatment (Fig. 1A). The CX3CL1 protein content in AML12 and 603B cell lyses was measured by ELISA (Fig. 1B). CX3CL1 protein content in AML12 cell lyses started to increase after IFN-γ treatment for 4 h and reached to the peak at 12 h, then declined after 12 h following IFN-γ treated (Fig. 1B). Similarly, we found that the CX3CL1 protein level in 603B cells was increased at 4 h and started to decrease at 24 h after IFN-γ treatment (Fig. 1B). These data suggested that IFN-γ stimulation induces only a transient CX3CL1 expression in liver epithelial cells.

### IFN-γ stimulation decreases CX3CL1 mRNA stability

**Figure 2:**
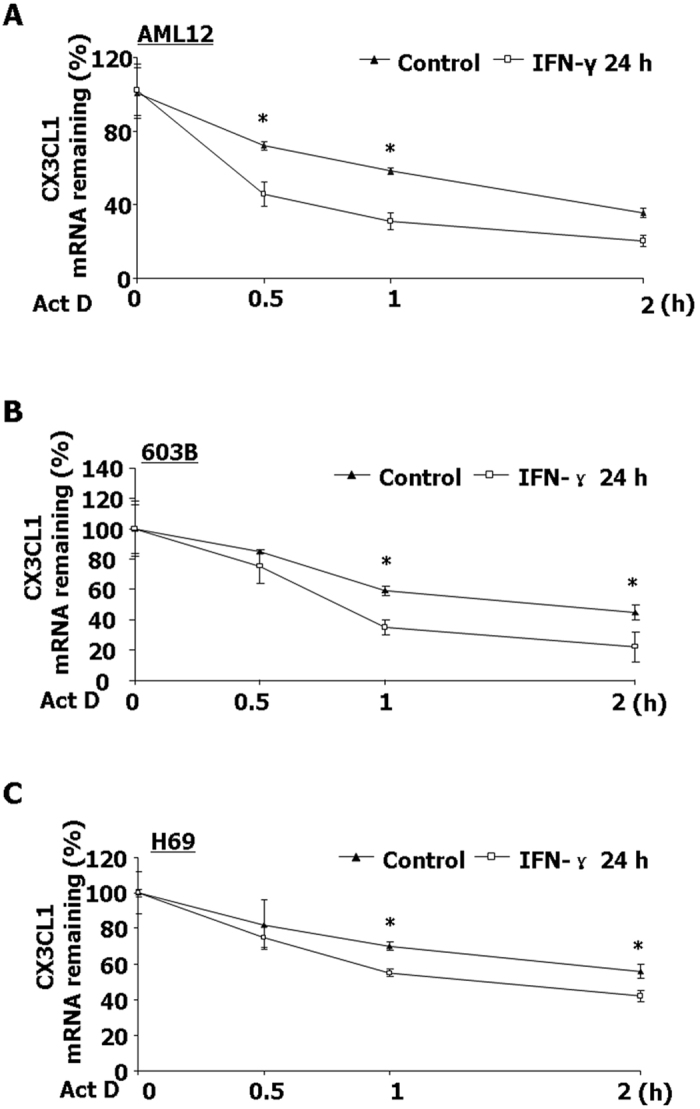
IFN-γ stimulation decreases CX3CL1 mRNA stability. AML12 (**A**); 603B (**B**) and H69 cells (**C**) were stimulated by IFN-γ (10 ng/ml) for 24 h. Actinomycin D (Act **D**) was then added and cells were collected for real-time PCR analysis. The stability of mRNAs was calculated, presented as the relative amount of mRNA to cells (Control) following IFN-γ stimulation for another 2 h before Act D treatment. Quantification of CX3CL1 mRNA levels from three independent experiments was shown. *p < 0.05 vs. non-pretreated cells.

To assess the potential effects of IFN-γ stimulation on the stability of CX3CL1 mRNA, AML12, 603B and H69 cells were treated with IFN-γ for 24 h. Cells were then treated with actinomycin D in the presence of IFN-γ for additional 2 h, followed by measurement of CX3CL1 mRNA by real-time PCR. After actinomycin D treatment, the decay of CX3CL1 mRNA in the IFN-γ-treated cells was faster, compared with that for the untreated cells (Fig. 2A–C). To test whether IFN-γ treatment influence mRNA stability of other pro-inflammatory effectors, we tested mRNA stability of COX-2 and iNOS in AML12, H69 and 603B cells following IFN-γ stimulation. Cells pre-treated with IFN-γ also displayed decreased mRNA stability of COX-2 and iNOS compared with the untreated cells (Fig. S1A–S1C).

### IFN-γ stimulation induces expression of KSRP protein without change in KSRP mRNA *in vitro* and *in vivo*

**Figure 3:**
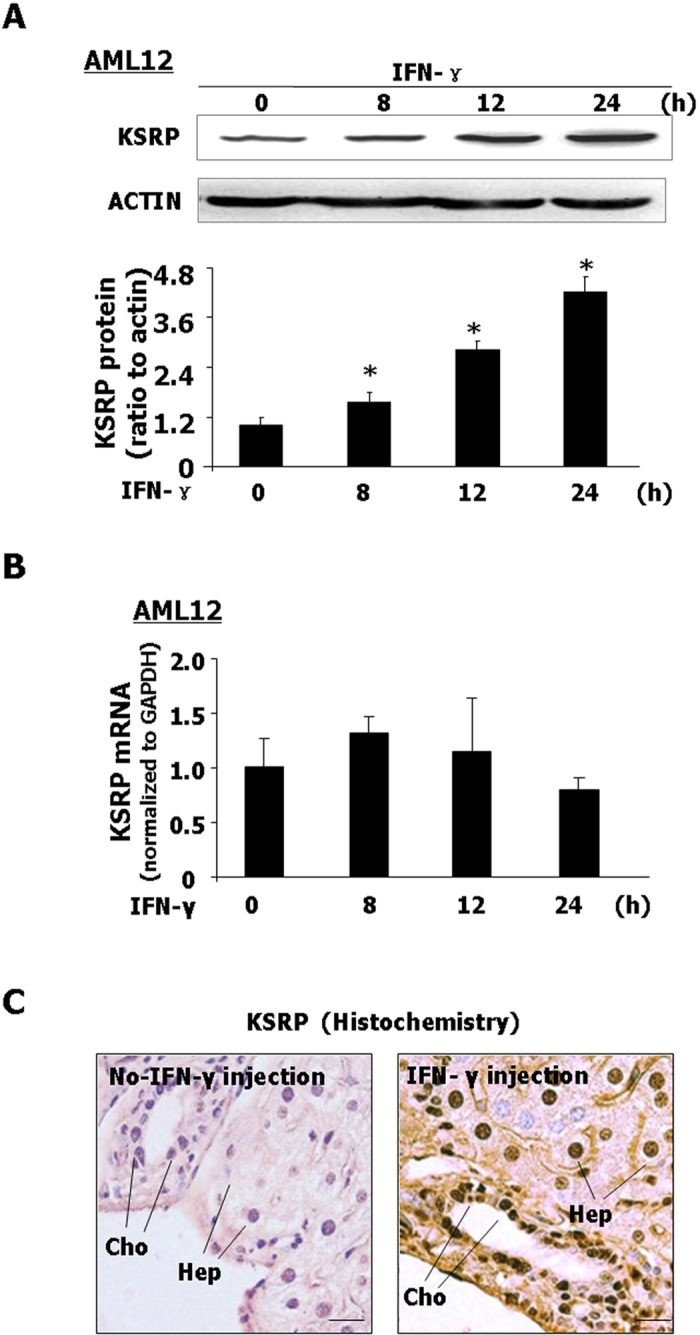
IFN-γ stimulation induces expression of KSRP protein without change in KSRP mRNA *in vitro* and *in vivo*. (**A,B**) AML12 cells were exposed to IFN-γ (10 ng/ml) for up to 24 h, followed by Western blot for KSRP proteins and real-time PCR analysis for KSRP mRNA. Representative Western blot gels and quantification of KSRP mRNA levels from three independent experiments are shown. Densitometric levels of KSRP signals were quantified and expressed as the ratio to actin. *p < 0.05 vs. non-treated cells. (**C**) Expression of KSRP protein staining was detected by immunohistochemistry in hepatocytes and cholangiocytes from mice (24 h) following IFN-γ i.p. injection (Bar = 10 nm).

Destabilization of CX3CL1 mRNA in cells following IFN-γ stimulation, along with the mRNAs of COX-2 and iNOS, suggests to us that a similar mechanism may be involved. KSRP is a RNA binding protein, which can recognize AREs within the 3′UTRs of mRNAs of COX-2, iNOS and control their half-life time in the cytoplasm^11^. The 3′UTR of CX3CL1 mRNA also possesses the ARE sequence^21^. We then assessed KSRP expression at the mRNA level and protein level in hepatocytes and biliary epithelial cells in response to IFN-γ stimulation. The expression of KSRP protein started to increase in AML12 cells after IFN-γ treated for 8 h and continue to increase till 24 h following IFN-γ stimulation (Fig. 3A). The increased of KSRP at protein level was also detected in H69 and 603B cells (Fig. S2A). A dose-dependent increase of KSRP protein expression was detected by Western blot in H69 and 603B cells following IFN-γ stimulation (Fig. S2A). Interestingly, no significant change of KSRP mRNA levels was found by real-time PCR in IFN-γ-treated AML12 cells (Fig. 3B), H69 cells, 603B cells and primary hepatocytes isolated from mice (Fig. S2B). Consistent with *in vitro* results, a significant increase of KSRP protein content was detected in the liver from mice after IFN-γ i.p. injection for 24 h by immunohistochemistry (Fig. 3C). Consistent with results of previous studies^22^, we observed that KSRP protein was strongly enriched in the nuclei in hepatocytes and biliary epithelial cells after IFN-γ i.p. injection (Fig. 3C).

### IFN-γ stimulation decreases miR-27b expression *in vitro* and *in vivo*

**Figure 4:**
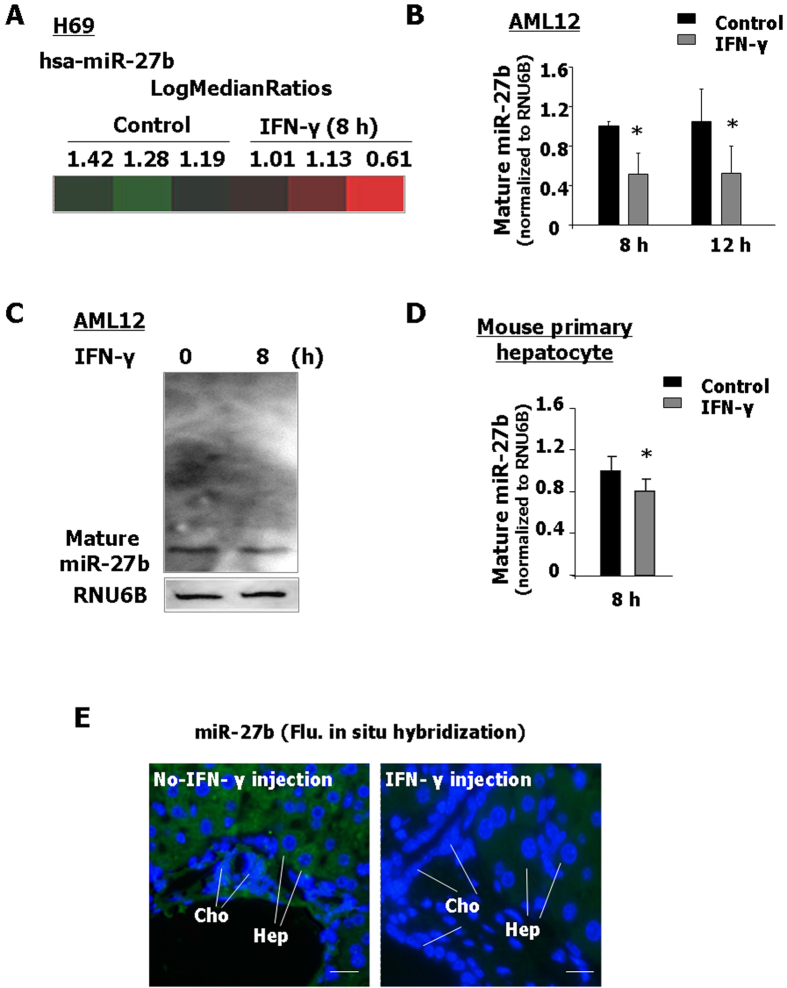
IFN-γ stimulation decreases miR-27b expression *in vitro* and *in vivo*. (**A–C**) H69 and AML12 cells were exposed to IFN-γ (10 ng/ml) for 8 h or 12 h, followed by microarray (**A**), real-time PCR (**B**) and Northern blot (**C**) analysis for miR-27b. (**A**) Levels of miR-27b by microarray are shown in a heat-map and presented as the LogMedianRatios. (**B**) Alterations in mature miR-27b expression in AML12 cells following IFN-γ (10 ng/ml) infection. Cells were exposure to IFN-γ (10 ng/ml) for 8 h and 12 h and the level of mature miR-27b was measured by real-time PCR, normalizing to RNU6B and relative to the non-treated cells. (**C**) AML12 cells were exposed to IFN-γ (10 ng/ml) for 8 h by Northern blot for miR-27b. (**D**) Alterations in mature miR-27b expression in Mouse primary hepatocyte cells following IFN-γ infection. Cells were exposure to IFN-γ (10 ng/ml) for 8 h and the level of mature miR-27b was measured by real-time PCR, normalizing to RNU6B and relative to the non-treated cells. (**E**) Expression of miR-27b in hepatocytes and biliary epithelial cells from mice in the presence or absence of IFN-γ injection for 24 h. miR-27b was labeled in green by *in situ* hybridization using a DIG-conjugated probe and cell nuclei by DAPI in blue (Bar = 10 nm). Representative Northern blot gels from three independent experiments are shown. *p < 0.05 t-test vs. non-treated cells (in **B**,**D**).

Given the detection of an increased amount of KSRP protein without a significant change in its mRNA levels in cells following IFN-γ stimulation, we expected that miRNA-mediated post-transcriptional regulation may be involved. We previously described an altered expression profile of mature miRNAs in human biliary epithelial cells following IFN-γ stimulation^23^. Of these miRNAs expressed in H69 cells, expression of miR-27b showed a tendency to decrease (0.05 < p < 0.10) after exposure to IFN-γ for 8 h by the miRCURYTM LNA human microRNAs assays (Fig. 4A). The expression of mature miR-27b was showed decreased in AML12 cells after IFN-γ treatment for 8 h and 12 h by real-time PCR (Fig. 4B). A decrease of miR-27b expression was also observed in H69 and 603B cells following IFN-γ stimulation for 8 h (Fig. S3). Our Northern blot analysis further confirmed the decrease of mature miR-27b in AML12 cells following IFN-γ stimulation for 8 h (Fig. 4C). Of note, pre-miR-27b was not obvious in the Northern gel, suggesting a low level of the primary transcript of miR-27b in the cells. Moreover, the expression of miR-27b was decreased in primary mouse hepatocytes after IFN-γ treatment for 8 h (Fig. 4D). Our *in situ* hybridization demonstrated a predominant cytoplasmic labeling for mature miR-27b in both hepatocytes and biliary epithelial cells of the liver tissues from non-IFN-γ-treated mice (Fig. 4E). Nuclear labeling was not obvious, representing a low level of primary transcript in the nuclei and consistent with the results from our Northern Blot analysis on AML12 cells. A lower level of miR-27b expression signal was detected in hepatocytes and biliary epithelial cells in IFN-γ-injected mice by *in situ* hybridization, compared to the untreated mice (Fig. 4E).

### KSRP regulates the stabilization of CX3CL1 mRNA through targeting ARE

**Figure 5:**
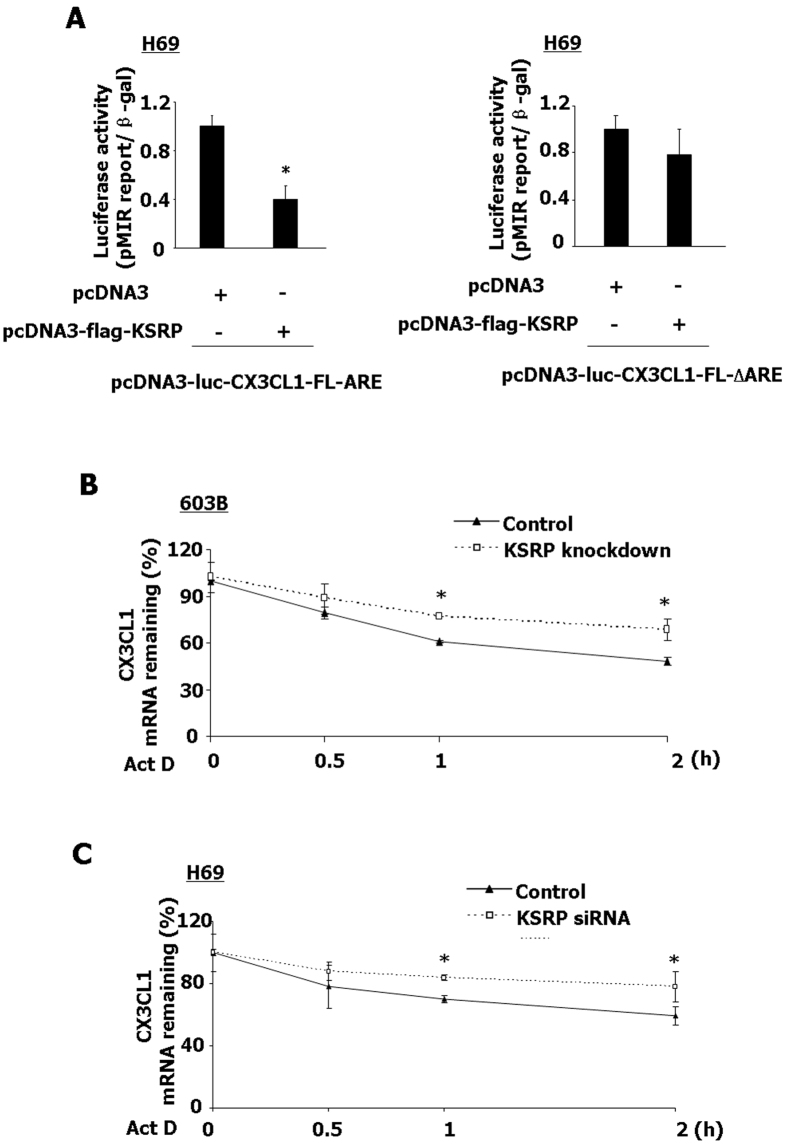
KSRP regulates the stabilization of CX3CL1 mRNA through targeting ARE. (**A**) KSRP decreased CX3CL1 ARE-associated luciferase activity. H69 cells were co-transfected with the luciferase construct containing the ARE in full-length CX3CL1 3′UTR (pcDNA3-luc-CX3CL1-FL-ARE) or the CX3CL1 3′UTR with deletion of ARE (pcDNA3-luc-CX3CL1-FL-△ARE), and pcDNA3-flag-KSRP for 24 h, followed by luciferase analysis. B and C: Effects of KSRP knockdown on CX3CL1 mRNA stability in 603B (**B**) and H69 (**C**) cells. The stability of CX3CL1 mRNAs was calculated in 603B cells stably expressing KSRP shRNA (**B**) or in H69 cells transient transfected by KSRP siRNA (**C**), and both 603B and H69 cells following IFN-γ stimulation for another 2 h. Representative quantification of luciferase activity and KSRP mRNA levels from three independent experiments are shown. *p < 0.05 t-test vs. the control.

Both human and mouse CX3CL1 mRNA 3′UTR contain a single UUAUUUAUU nonamer^21^. To determine whether KSRP plays a role in the regulation of CX3CL1 expression through targeting ARE, we used two constructs containing the luciferase cDNA fused to the full-length CX3CL1 3′UTR (pcDNA3-luc-CX3CL1-FL-ARE) and the full-length CX3CL1 3′UTR with deletion of ARE (pcDNA3-luc-CX3CL1-FL-△ARE). Two constructs containing the truncated 3′UTR containing a UUAUUUAUU nonamer (pcDNA3-luc-CX3CL1-ARE) and deletion of the UUAUUUAUU nonamer of 3′UTR (pcDNA3-luc-CX3CL1-▵ARE) were also used in the experiments. H69 cells were transfected with these constructs with pcDNA3-flag-KSRP (overexpression of KSRP) by assessment of luciferase activity 24 h after transfection. The transfection efficiency for the luciferase plasmids in H69 cells was around 50% and therefore, luciferase activity was normalized to the expression of the control β-gal construct. Overexpression KSRP in H69 cells resulted in a significant decrease in luciferase activity of pcDNA3-luc-CX3CL1-FL-ARE and pcDNA3-luc-CX3CL1-ARE, compared with that in the control (Fig. 5A and S4, respectively). In cells transfected with the construct deletion of the ARE nonamer, the associated luciferase activity was not changed, compared with the control (Fig. 5A and S4, respectively). To further identify whether KSRP regulates CX3CL1 mRNA stability, we measured CX3CL1 mRNA stability in KSRP knockdown cells. Transfection of 603B cells with a mouse KSRP shRNA construct or treatment of H69 cells with a siRNA to human KSRP significantly inhibited the expression of KSRP mRNA (Fig. S5). As shown in Fig. 5B, 603B cells stably expressing KSRP shRNA displayed a significant increase in CX3CL1 mRNA stability. The increased mRNA stability of CX3CL1 was also detected in H69 cells transfected with KSRP siRNA compared to the control cells (Fig. 5C).

### miR-27b regulates the stabilization of CX3CL1 mRNA in an ARE-dependent manner

**Figure 6:**
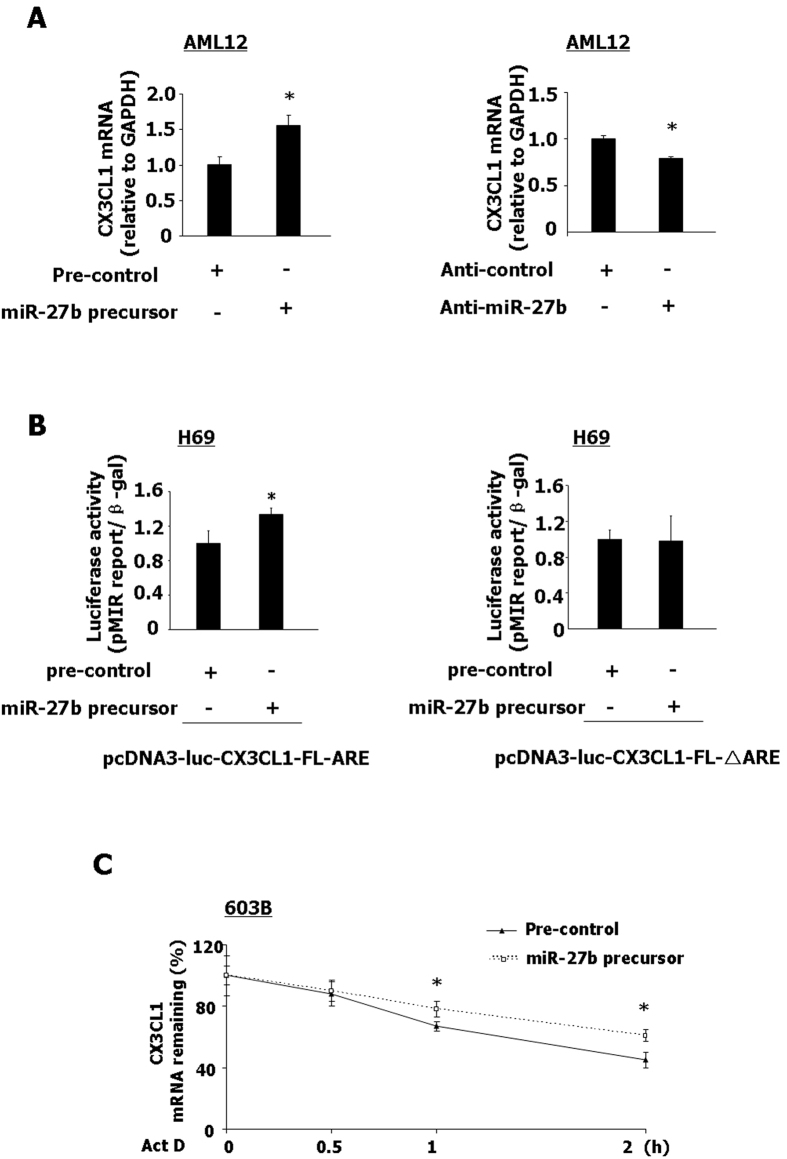
miR-27b regulates the stabilization of CX3CL1 mRNA in an ARE-dependent manner. (**A**) AML12 cells were transfected with miR-27b precursor or anti-miR-27b for 48 h, followed by real-time PCR for CX3CL1 mRNA. (**B**) H69 cells were transfected with the luciferase construct containing ARE in full-length CX3CL1 3′UTR (pcDNA3-luc-CX3CL1-FL-ARE) or the CX3CL1 3′UTR with deletion of ARE (pcDNA3-luc-CX3CL1-FL-△ ARE), and transfected with miR-27b precursor for 24 h, followed by luciferase analysis. (**C**) Effects of miR-27b precursor in CX3CL1 mRNA stability in 603B cells. 603B cells were transfected with miR-27b precursor or pre-control for 48 h. The stability of mRNAs was calculated in cells following IFN-γ stimulation for another 2 h. Representative quantification of luciferase activity and CX3CL1 mRNA levels from three independent experiments was shown. *p < 0.05 t-test vs. the control.

Our previous study suggested that miR-27b can target KSRP in epithelial cells^18^. We then asked whether miR-27b regulates CX3CL1 mRNA stability. CX3CL1 mRNA expression was measured in AML12 cells transfected with anti-miR-27b or miR-27b precursor. miR-27b precursor increased CX3CL1 mRNA expression compared with the control (Fig. 6A). In contrast, anti-miR-27b showed a significant decrease in the expression of CX3CL1 mRNA (Fig. 6A). To determine whether miR-27b regulates CX3CL1 expression through targeting ARE in 3′UTR, H69 cells were co-transfected with constructs containing CX3CL1 3′UTR (pcDNA3-luc-CX3CL1-FL-ARE or pcDNA3-luc-CX3CL1-ARE) or the CX3CL1 3′UTR with deletion of ARE (pcDNA3-luc-CX3CL1-FL-△ARE or pcDNA3-luc-CX3CL1-△ARE ) together with the miR-27b precursor. miR-27b precursor transfection showed a significant increase of luciferase activity in H69 cells transfected with constructs containing CX3CL1 3′UTR with ARE (pcDNA3-luc-CX3CL1-FL-ARE and pcDNA3-luc-CX3CL1-ARE), compared to the control (Fig. 6B and S6, respectively). Inhibition of associated luciferase activity was not detected in cells transfected with the ARE-deleted construct, compared to the control after miR-27b precursor transfection (Fig. 6B and S6, respectively). Furthermore, CX3CL1 mRNA stability was measuring in 603B cells transfected with miR-27b precursor, comparing with the control (Fig. 6C). As shown in Fig. 6C, 603B cells transfected with miR-27b precursor displayed a significant increase in CX3CL1 mRNA stability.

### IFN-γ stimulation destabilized CX3CL1 mRNA through targeting ARE

**Figure 7:**
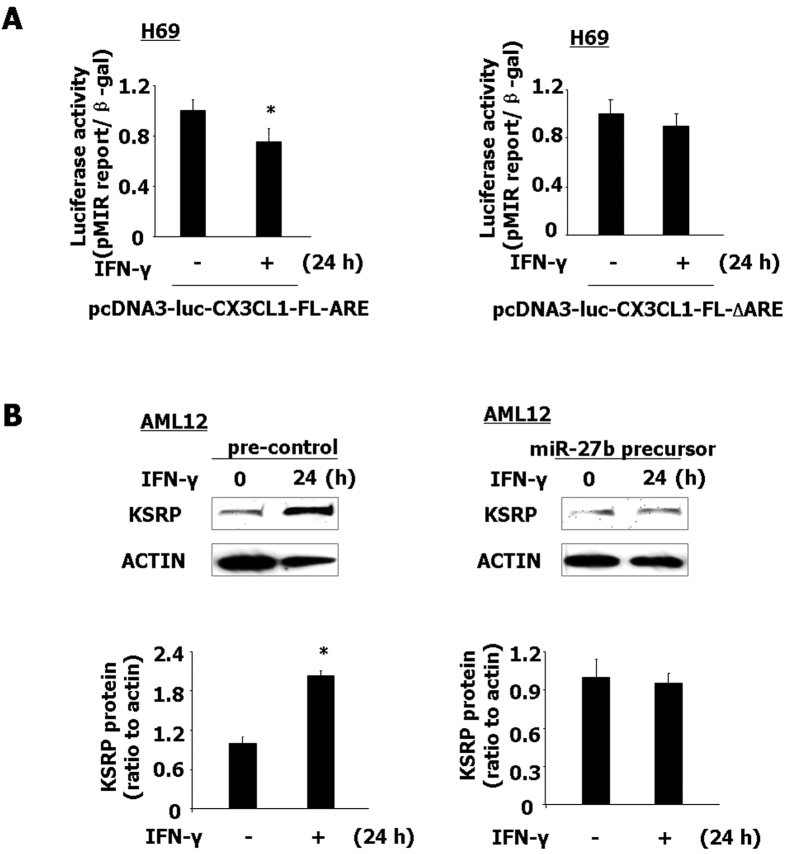
IFN-γ stimulation destabilizes CX3CL1 mRNA through targeting ARE. (**A**) IFN-γ stimulation decreased ARE of CX3CL1-associated luciferase activity. H69 cells were transfected with the luciferase construct containing ARE in full-length CX3CL1 3′UTR (pcDNA3-luc-CX3CL1-FL-ARE) or the CX3CL1 3′UTR with deletion of ARE (pcDNA3-luc-CX3CL1-FL-△ARE), and stimulated by IFN-γ for 24 h, followed by luciferase analysis. (**B**) miR-27b precursor inhibited upregulated of KSRP protein induced by IFN-γ stimulation. AML12 cells were transfected with miR-27b precursor or pre-control for 48 h and then exposed to IFN-γ for 24 h, followed by Western blot for KSRP protein. Representative quantification of luciferase activity and protein levels from three independent experiments was shown. Densitometric levels of KSRP signals were quantified and expressed as the ratio to actin. *p < 0.05 t-test vs. the control (**A**); *p < 0.05 vs. non-treated cells (**B**).

Given the downregulation of miR-27b after IFN-γ stimulation and the impact of miR-27b on CX3CL1 mRNA stability through targeting ARE, we speculated that IFN-γ treatment should affect ARE-mediated post-transcription of CX3CL1. To test this possibility, H69 cells were transfected with constructs containing CX3CL1 3′UTR with ARE (pcDNA3-luc-CX3CL1-FL-∆ARE or pcDNA3-luc-CX3CL1-∆ARE) or the CX3CL1 3′UTR with deletion of ARE (pcDNA3-luc-CX3CL1-FL-∆ARE or pcDNA3-luc-CX3CL1-∆ARE) and then exposed to IFN-γ stimulation for 24 h. IFN-γ treatment decreased the luciferase activity in H69 cells transfected constructs containing CX3CL1 3′UTR with ARE, but did not alter the luciferase activity in cells transfected with constructs containing deletion of ARE (Fig. 7A and S7, respectively). In addition, AML12 cells were transfected with miR-27b precursor for 24 h, and then exposed to IFN-γ for another 24 h, followed by Western blot for KSRP protein. miR-27b precursor inhibited the upregulation of KSRP protein in AML12 cells induced by IFN-γ stimulation (Fig. 7B). Taken together, the above data suggest that miR-27b regulates the stabilization of CX3CL1 mRNA through targeting ARE following IFN-γ stimulation.

### miR-27b regulates chemotaxis effects of CX3CL1 to Jurkat cells

**Figure 8:**
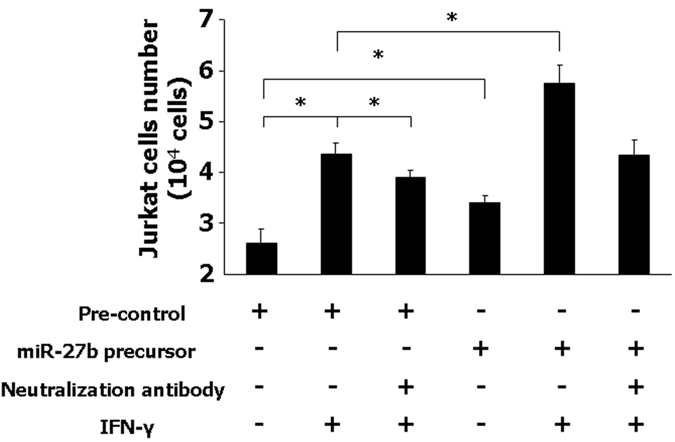
miR-27b regulates chemotaxis effects of CX3CL1 to Jurkat cells. H69 cells were transfected with miR-27b precursor for 48 h, then exposed to IFN-γ for 24 h in the presence or absence of a neutralizing Ab to CX3CL1. H69 cells were then cocultured with Jurkat cells. Number of Jurkat cells transmigrated in cell chambers after 2.5 h was counted. Normal rabbit IgG was used as a negative control. *p < 0.05 t-test vs. the control.

Membrane-bound CX3CL1 has been reported to function as an adhesion molecule to interact with CX3CR1-positive immune cells, including T-cells, NK cells, and monocytes^13^. We then tested the role for CX3CL1-induced chemotaxis activity in liver epithelial cells. Because of the potential histocompatibility between different species, we chose to use a co-culture system employing two human cell lines: Jurkat cells in 8 nm membrane inserts co-cultured with IFN-γ-treated H69 cells. The JKT is a cancer cell line of leukemia with certain changes of the original T cell characteristics^24^. However, it expresses CX3CR1 and has previously been used for chemotaxis assay^25^. H69 cells were treated with IFN-γ after miR-27b precursor transfection in the presence or absence of a neutralizing Ab to CX3CL1. After 2.5 h of incubation, Jurkat cells migrated through the membrane into the H69 medium pool were counted. A significant increase of Jurkat cell migration was detected after IFN-γ treatment (Fig. 8). Neutralizing Ab to CX3CL1 showed a significant inhibitory effect of Jurkat cell migration induced by IFN-γ. Moreover, miR-27b precursor transfection showed a significant increase of Jurkat cell migration after exposed to IFN-γ (Fig. 8). IFN-γ-stimulated Jurkat cell migration was partially blocked after miR-27b precursor transfection of H69 cells, suggesting that miR-27b regulates chemotaxis effects of CX3CL1-expressing H69 cells to Jurkat cells.

## Discussion

Our findings reveal a novel role for miR-27b in the negative regulation of immune reactions in liver epithelial cells in response to IFN-γ stimulation. We found that IFN-γ stimulation decreased miR-27b expression and increased KSRP protein content without changing its mRNA level in liver epithelial cells *in vitro* and *in vivo.* Besides these traditional KSRP-regulated ARE-containing mRNAs of iNOS and COX-2^18^, upregulation of KSRP destabilized CX3CL1 mRNA through interacting with the ARE within its 3′UTRs in liver epithelial cells. Through targeting KSRP, miR-27b regulated the stabilization of CX3CL1 mRNA. Consequently, downregulation of miR-27b following IFN-γ stimulation resulted in KSRP induction, providing negative feedback regulation of cytokine/chemokine expression in liver epithelial cells.

Persistent inflammation is mediated by increased expression of multiple pro-inflammatory mediators in most chronic inflammatory diseases. The coordinated expression of the protein components of a complex functional program such as the inflammatory response involves both transcriptional and post-transcriptional mechanisms^6^. A central part of post-transcriptional modulation of gene expression is mediated by the regulation of mRNA stability. A tight control of mRNA stability permits rapid changes in the levels of mRNAs and provides a mechanism for prompt termination of protein production^7^,^8^. The mRNAs encoding subsets of inflammation-relative proteins potentially injurious to host tissues, such as iNOS and COX-2, contain regulatory elements that direct their degradation and translational repression to protect against pathological overexpression^9^,^10^,^18^. Our study provides a new mechanism of controlling excessive inflammation through downregulation of miR-27b to modulate KSRP expression.

Previous study indicated that CX3CL1 plays an important role in chronic inflammatory diseases^26^. The expression of CX3CL1 is up-regulated in chronic liver diseases such as chronic hepatitis C^16^. Transcriptional and post-transcriptional regulations were both involved in the regulation of CX3CL1 expression^21^. The 3′UTR of CX3CL1 mRNA contains AREs and HuR has been shown to modulate CX3CL1 mRNA stability by binding to the AREs within the 3′UTR and to control its half-life time in the cytoplasm^21^. In this study, for the first time, we demonstrated that CX3CL1 mRNA stability is directly regulated by KSRP through its interaction with the AREs within CX3CL1 mRNA 3′UTR. This post-transcriptional mechanism is involved in the transient expression of CX3CL1 in liver epithelial cells in response to IFN-γ stimulation.

KSRP is a single-strand nucleic acid binding protein and known to be one of the most important mRNA destabilizing proteins^22^. Some of these KSRP-regulated mRNAs code proteins with important immune functions, such as iNOS, and COX-2^11^,^27^, and now, include CX3CL1. Given its critical role in regulating inflammatory responses, KSRP should be tightly controlled in the cells at physiological conditions. Previous studies demonstrated that at least two signaling pathways, the MAPK p38 and the Akt/PKB, can target KSRP and modulate ARE-mediated mRNA decay^28^. In our previous studies, we reported that miR-27b targets KSRP 3′UTR resulting in translational suppression of KSRP in biliary epithelial cells^18^. Upregulation of miR-27b suppresses KSRP expression, facilitating NO production through stabilization of iNOS in biliary epithelial cells against *C. parvum* infection^18^. Here, our findings indicate that IFN-γ stimulation downregulates miR-27b expression in liver epithelial cells, providing negative feedback regulation of cytokine/chemokine expression through KSRP induction in the liver.

miRNA-mediated post-transcriptional mechanisms have recently been demonstrated to play an important role in regulation of epithelial innate immunity^29^. Recent evidence showing altered miRNA expression in various inflammation cells suggested their involvement in inflammatory diseases^2^. Importantly, alterations in miRNA expression following stimulation are controlled by activation of intracellular signaling pathway network. We previously demonstrated that *C. parvum* infection and LPS stimulation activates liver epithelial cell TLR4/NF-ĸB signaling, resulting in alterations in expression of a panel of miRNAs^18^,^30^. In this regard, how IFN-γ stimulation suppresses the expression of miR-27b is still unclear and merits further investigation. Our findings cannot exclude the possibility of miRNA targeting of CX3CL1 mRNA 3′UTR, resulting in post-transcriptional regulation of CX3CL1 expression in liver epithelial cells.

In summary, our study provides evidence that miR-27b regulates the mRNA stability of CX3CL1 to maintain cellular homeostasis through targeting KSRP in liver epithelial cells. Post-transcriptional regulation of CX3CL1 stability by KSRP through miRNAs may represent a new mechanism by which liver epithelial cells maintain homeostasis during inflammation, relevant to fine-tuning of cellular inflammatory responses in general.

## Materials and Methods

### Ethics statement

Male C57BL/6J mice, 6 to 8 week old, weighing 22–25 g were used. Animal protocols were approved by the Animal Care and Use Committee of Wuhan University and all experiments were performed in accordance with the guidelines and regulations of the university.

All surgeries were performed under ketamine (100 mg/kg body weight, i.p) and xylazine (10 mg/kg body weight, i.p.) anesthesia, and all efforts were made to minimize suffering to the animals.

### Cell lines

H69 cells (a gift of Dr. D. Jefferson, Tufts University) are SV40 transformed normal human biliary epithelial cells originally derived from liver harvested for transplant. These cells continue to express biliary epithelial cell markers, including cytokeratin 19, gamma glutamyl transpeptidase and ion transporters consistent with biliary function and have been extensively characterized^31^. The 603B cells are immortalized normal mouse biliary epithelial cells (a gift from Y. Ueno, Tohoku University School of Medicine, Sendai, Japan). AML12 is a murine hepatocyte cell line and was obtained from the American Type Culture Collection (CD1 strain, line MT42). Primary murine hepatocytes were purchased from Celsis (Chicago, USA) and cultured according to the instructions from the company.

### Plasmids and reagents

The expression vector for KSRP (pcDNA3-Flag-KSRP) carrying insertion of the coding regions of KSRP is a kind gift from Dr. Ching-Yi Chen (University of Alabama, Birmingham, AL). A KSRP shRNA construct was prepared in the vector pRNA-U6.1/hygro. The target sequence for KSRP was based on sequences within the KSRP coding region (GGACAGTTTCACGACAACG). Human KSRP siRNA purchased from Santa Cruz Biotechnology. The vectors containing human CX3CL1 ARE of 3′UTR or CX3CL1 3′UTR with deletion of AU-rich element (pcDNA3-luc-CX3CL1-FL-ARE;pcDNA3-luc-CX3CL1–ARE; pcDNA3-luc-CX3CL1-FL-△ARE and pcDNA3-luc-CX3CL1-△ARE) are kind gifts from Dr. Matsumiya (Hirosaki University Graduate School of Medicine, Hirosaki City, Japan). Actinomycin D (10 μg/ml) was purchased from Fisher Scientific (Pittsburgh, PA). At the utilized concentrations, no cytotoxic effects of any of the chemicals were observed on AML12, H69 and 603B cells (data not shown).

### Real-time PCR

For real-time PCR analysis of mature miRNAs, total RNAs were extracted using the mirVana™ miRNA Isolation kit (Applied Biosystems). An amount of 0.05 μg total RNAs was reverse-transcribed by using the Taqman MicroRNA Reverse Transcription Kit (Applied Biosystems). Comparative real-time PCR was performed in triplicate using Taqman Universal PCR Master Mix (Applied Biosystems) on the Applied Biosystems 7500 FAST real-time PCR System. Mature miR-27b primers and probes were obtained from Applied Biosystems. Normalization was performed by using RNU6B primers and probes. Relative expression was calculated by using the comparative CT method^30^,^32^.

For analysis of mRNA, total RNA was isolated from cells with Trizol reagent (Applied Biosystems). RNAs were treated with DNA-free^TM^ Kit (Applied Biosystems) to remove any remaining DNA. Comparative real-time PCR was performed by using the SYBR Green PCR Master Mix (Applied Biosystems). Specific primers for mRNAs were listed in Table S1. All reactions were run in triplicate. The Ct values were analyzed using the comparative Ct (△△Ct) method and the amount of target was obtained by normalizing to the endogenous reference and relative to the control (non-treated cells)^32^.

### ELISA

For the measurement of CX3CL1 production by AML12 and 603B cells in six well culture plates were stimulated with IFN-γ (10 ng/ml) for up to 48 h. Cells were collected with lysis buffer at different time point following IFN-γ stimulation. The level of CX3CL1 in the cell lyses was determined using a Quantikine ELISA kit (R&D Systems).

### Western blot

Whole cell lysates were obtained from cells with MPER mammalian protein extraction reagent (Pierce) containing several protease inhibitors (1 mM PMSF, 10 μg/ml leupeptin, and 2 μg/ml pepstatin). Cell lysates were then loaded in SDS-PAGE gel to separate proteins and transferred to nitrocellulose membrane. Antibodies to KSRP (Bethyl Laboratories) and actin (Sigma-Aldrich) were used. Densitometric levels of Western blot signals were quantified and expressed as their ratio to actin^18^.

### IFN-γ injection *in vivo* and immunohistochemistry for KSRP

The C57BL/6J mice (The Jacksons Laboratory) were used for the study, with the approval of the Animal Care and Use Committee of Wuhan University. Animals received treatment of IFN-γ (5 μg in 200 μl of saline) by intraperitoneal (i.p.) injection for 24 h, as previously reported^33^. Five animals from each group were sacrificed and liver tissues obtained for immunohistochemistry. Antibodies to KSRP (Bethyl Laboratories) were utilized.

### RNA stability

Cells were stimulated by IFN-γ for 24 h or transfected with miR-27b precursor (30 nM) or KSRP siRNA (50 nM), then treated with IFN-γ (10 ng/ml) for another 2 h. Transcription was stopped by actinomycin D (10 μg/ml) and RNAs were prepared at various time points following actinomycin D treatment. Real-time PCR was then performed using 500 ng of template cDNA from the resultant RNA. Each sample was run in triplicate. The relative abundance of each mRNA was calculated using the ΔΔCt method and normalized to GAPDH (human) or actin (mouse). The relative amount of mRNA at 0 h following actinomycin D treatment was arbitrarily set to 1. Curve fittings of the resultant data were performed using Microsoft Excel and the half-lives of selected RNAs calculated, as previously reported^30^.

### *In situ* hybridization

Paraffin tissue sections were deparaffinized and treated with 10 μg/ml proteinase K (Roche) at 37 °C for 10 min, as reported^18^. After washing with PBS, slides were incubated with the hybridization buffer (50% formamide, 100 μg/ml salmon sperm DNA, 200 μg/ml yeast tRNA, 600 mM NaCL, 1 × Denhardt’s solution, 0.25% SDS, 1 mM EDTA) at 42 °C for 1 h. Slides were then hybridized with 20 nM DIG-labelled miR-27b probe (Exiqon) diluted in the hybridization buffer at 42 °C overnight. Slides were incubated with anti-DIG-POD Fab fragments (Roche) at 4 °C overnight and miR-27b was visualized in a staining reaction with Renaissance Tyramide Signal Amplification Fluorescence Systems (PerkinElmer). For all experiments, a negative control (i.e. staining without miR-27b probe) was included^18^.

### Transfection and Reporter assay

Transient transfections of H69 cells were accomplished by plating cells at a density of 1 × 10^5^cells per well of a 24-well culture plate 24 h before transfection. 250 ng of CX3CL1 3′UTR or its mutants and 250 ng pCMV-β-Gal were co-transfected using 1 μl of Lipofectamine 2000 transfection reagent (invitrogen) following the vendor’s instructions. The cells were incubated for 24 h and then analyzed for the reporter activity. Cells were harvested in Reporter lysis buffer (Progema), and the luciferase activity was determined by Luciferase assay system (Progema). Luciferase activities were normalized by transfection efficiency by β-gal activity measured using a β-gal reporter gene assay chemiluminescence kit (Roche, Basel, Switzerland). Data were presented as mean ± SD of at least three independent experiments.

### Chemotaxis assay

H69 and Jurkat cells were cocultured as previously reported^23^. Briefly, H69 cells were seeded at 1 × 10^5^ cells/well in 24-well plates, transfected with miR-27b precursor (30 nM) or pre-control (30 nM) for 48 h, then exposed to IFN-γ for 24 h in the presence or absence of a neutralizing Ab to CX3CL1 (Roche, Basel, Switzerland). H69 cells were then co-cultured with Jurkat cells grown in 8 nm membrane inserts (2 × 10^5^) as previously reported^25^. After 2.5 h of co-culture, Jurkat cells migrated through the insert membrane into the H69 medium pool were collected. The number of Jurkat cells was counted under a microscope as previously reported. Normal rabbit IgG (Santa Cruz) was used as a negative control.

## Additional Information

**How to cite this article**: Xia, Z. *et al.* Upregulation of KSRP by miR-27b provides IFN-γ-induced post-transcriptional regulation of CX3CL1 in liver epithelial cells. *Sci. Rep.* **5**, 17590; doi: 10.1038/srep17590 (2015).

## Supplementary Material

Supplementary Information
