# Supplementary material for: Upregulation of KSRP by miR-27b provides IFN-γ-induced post-transcriptional regulation of CX3CL1 in liver epithelial cells
Source: Sci Rep. 2015 Dec 3;5:17590. doi: 10.1038/srep17590 (PMC5009954; doi:10.1038/srep17590)
Supplement: Supplementary Information [file srep17590-s1.pdf]

Upregulation of KSRP by miR-27b provides IFN-γ-induced post-transcriptional regulation of CX3CL1 in liver epithelial cells

Zijie Xia[1#], Yajing Lu[1#], Xiaoqing Li[2], Tiebo Mao[1], Xian-Ming Chen[3], Rui Zhou[1*]

[1]School of Basic Medical Sciences, Wuhan University, Hubei 430071, China; [2]Institute of Hematology, Union Hospital, Tongji Medical School, Huazhong University of Science and Technology, Wuhan, Hubei 430000，China; [3]Department of Medical Microbiology and Immunology, Creighton University School of Medicine, Omaha, NE 68178, USA.

[#]These authors contributed equally to this work.

*Corresponding authors: Dr. Rui Zhou, School of Basic Medical Sciences, Wuhan University, 185 Donghu Road, Hubei 430071, China. E-mail: ruizhou@whu.edu.cn.

Figure S1. IFN-γ stimulation decreases COX-2 and iNOS mRNA stability.
AML12 (A); 603B (B) and H69 cells (C) were stimulated by IFN-γ (10 ng/ml) for 24 h. Actinomycin D (Act D) was then added and cells were collected for real-time PCR analysis. The stability of mRNAs was calculated, presented as the relative amount of mRNA to cells (Control) following IFN-γ stimulation for 2 h before Act D treatment. Quantification of COX-2 and iNOS mRNA levels from three independent experiments was shown. *, $p < 0.05$ vs. non-pretreated cells.

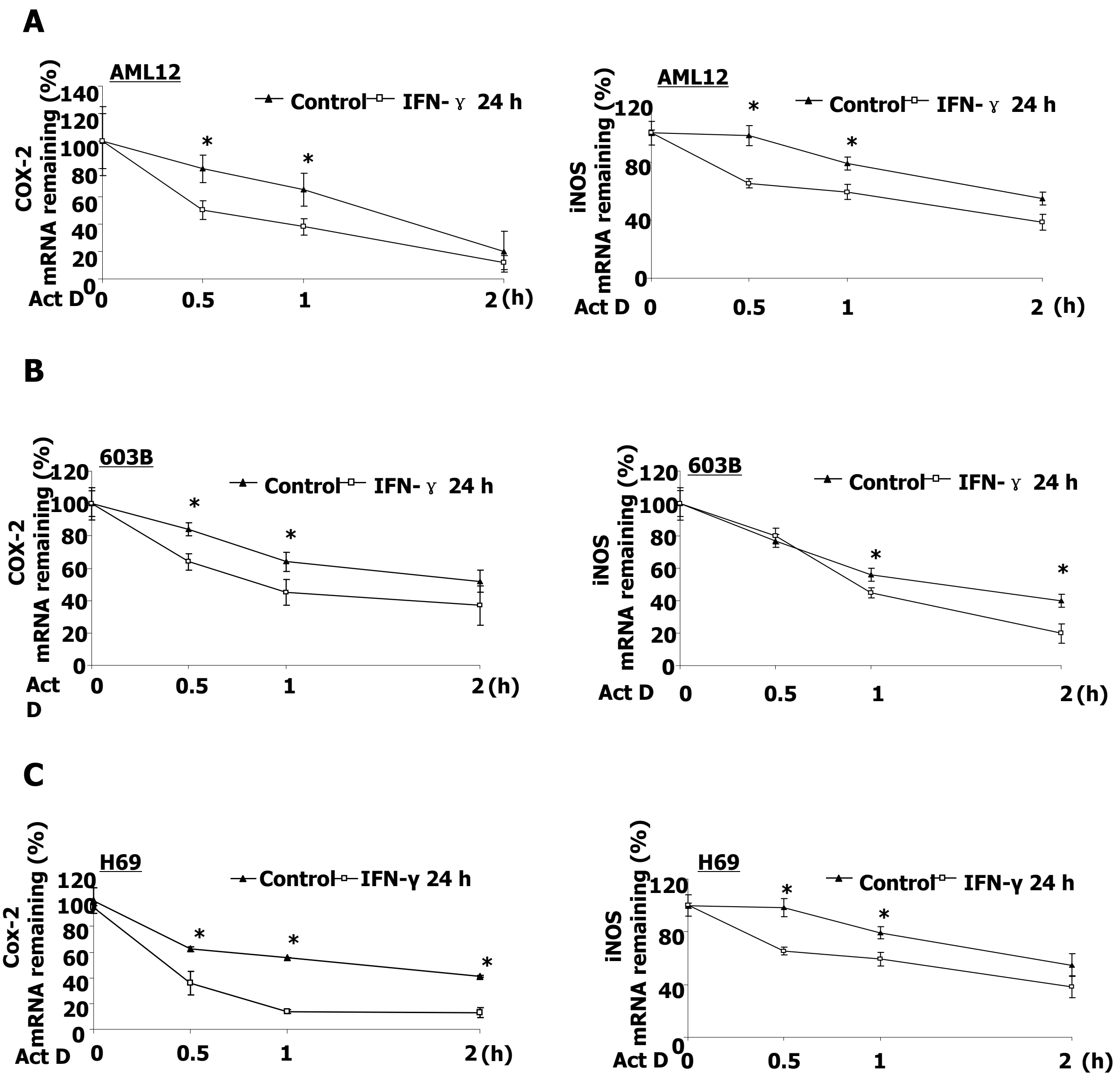

Figure S2. IFN-γ stimulation induces expression of KSRP protein without change in KSRP mRNA in 603B and H69 cells. A and B: 603B and H69 cells were exposed to IFN-γ for up to 24 h, followed by Western blot for KSRP proteins and real-time PCR analysis for KSRP mRNA. A dose-dependent and time-dependent upregulation of KSRP protein was detected following IFN-γ stimulation, whereas no significant change of KSRP mRNA level was detected in 603B, H69, and mouse primary hepatocyte cells. Representative Western blot gels and quantification of KSRP mRNA levels from three independent experiments was shown.

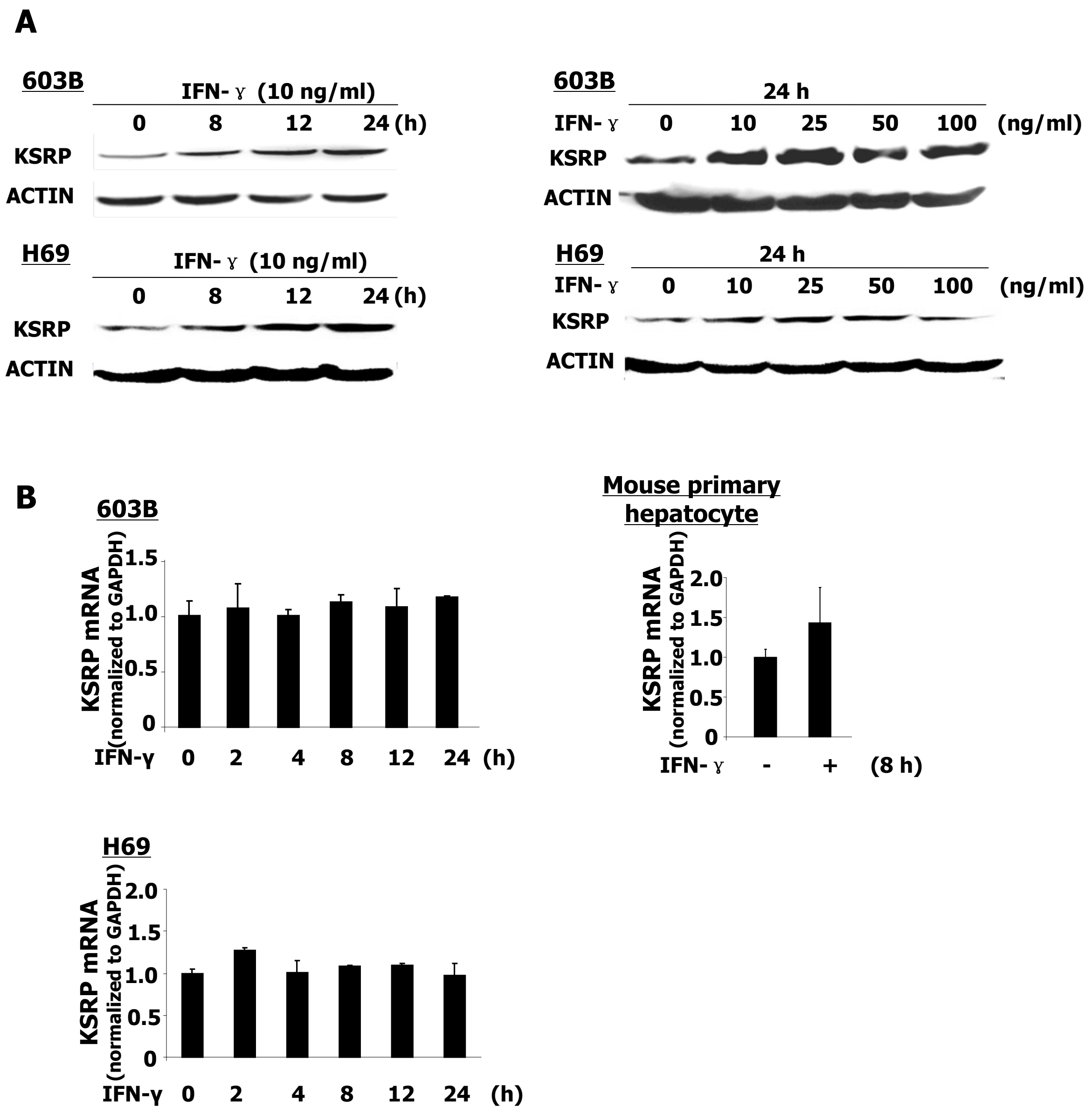

Figure S3. IFN-γ stimulation decreases miR-27b expression in H69 and 603B cells. Alterations of miR-27b expression after exposure to IFN-γ for 8 h were assessed by real-time PCR in 603B and H69 cells. The amount of mature miRNAs was obtained by normalizing to the level of RNU6B in the samples. Representative quantification of miR-27b levels from three independent experiments was shown. *, $p < 0.05$ vs. non-treated cells.

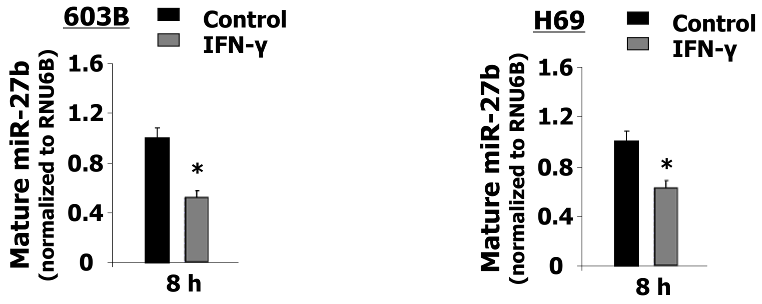

Figure S4. KSRP regulates the stabilization of CX3CL1 mRNA through targeting ARE. H69 cells were co-transfected with the luciferase construct containing the CX3CL1 ARE containing ARE in truncated CX3CL1 3' UTR (pcDNA3-luc-CX3CL1 -ARE) or truncated CX3CL1 3' UTR with deletion of ARE (pcDNA3-luc-CX3CL1-△ARE), and pcDNA3-flag-KSRP for 24 h, followed by luciferase analysis. Representative quantification of luciferase analysis from three independent experiments was shown. *, $p < 0.05$ t-test vs. the control.

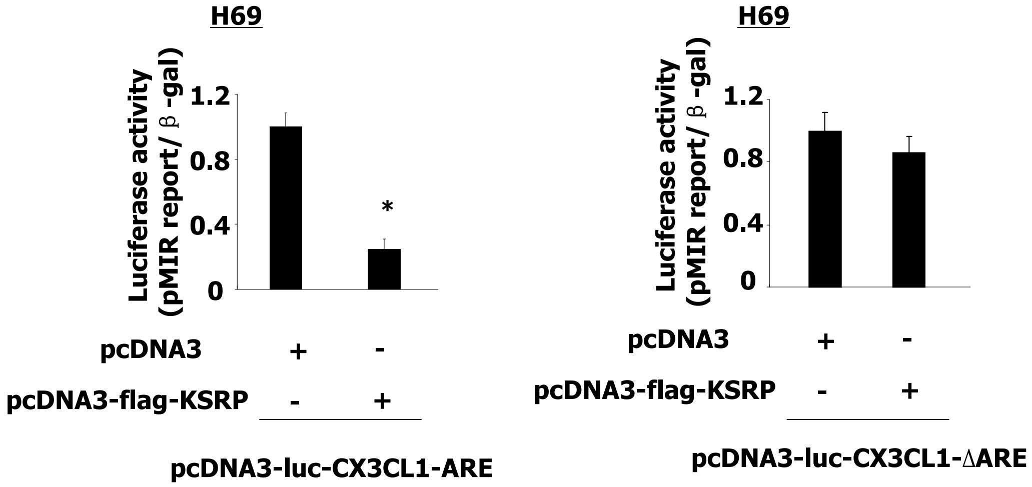

Figure S5. Effects of KSRP knockdown on KSRP mRNA expression in H69 and 603B cells. H69 cells transfected with KSRP siRNA and 603B cells stably expressing KSRP shRNA were selected by real-time PCR analysis for KSRP. Quantification of KSRP mRNA levels from three independent experiments was shown. *, $p < 0.05$ t-test vs. the control.

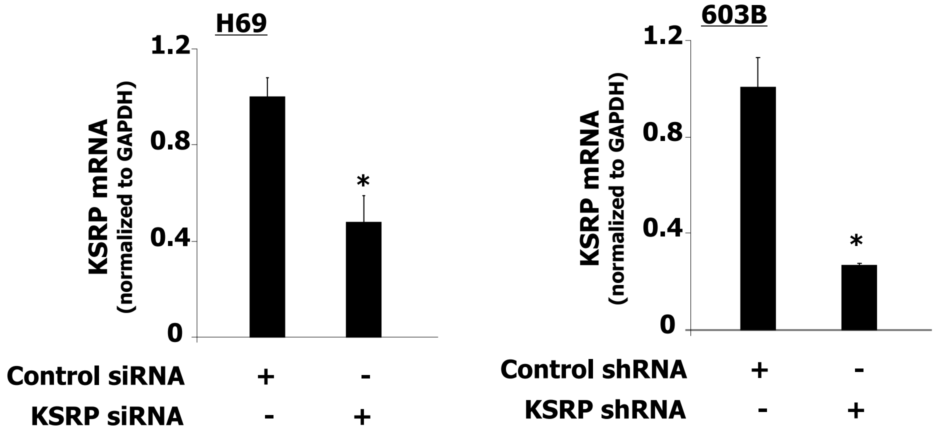

Figure S6. miR-27b regulates CX3CL1 ARE-associated luciferase activity.

H69 cells were transfected with the luciferase construct containing ARE in truncated CX3CL1 3’ UTR (pcDNA3-luc-CX3CL1-ARE) or truncated CX3CL1 3’ UTR with deletion of ARE (pcDNA3-luc-CX3CL1-△ARE), and treated with miR-27b precursor for 24 h, followed by luciferase analysis. Representative quantification of luciferase analysis from three independent experiments was shown. *, $p < 0.05$ t-test vs. the control.

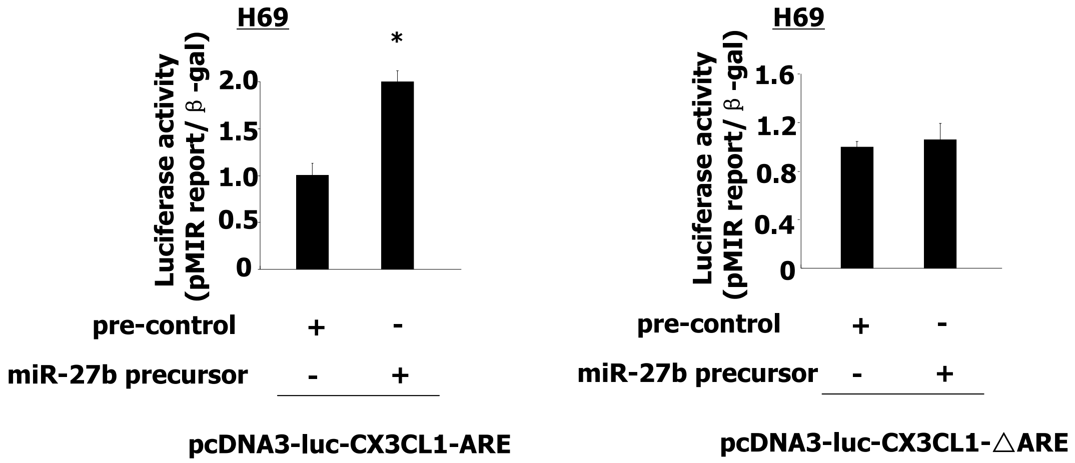

Figure S7. IFN-γ stimulation decreases CX3CL1 ARE-associated luciferase activity. H69 cells were transfected with the luciferase construct containing the ARE in truncated CX3CL1 3' UTR (pcDNA3-luc-CX3CL1-ARE) or truncated CX3CL1 3' UTR with deletion of ARE (pcDNA3-luc-CX3CL1-△ARE), then stimulated by IFN-γ for 24 h, followed by luciferase analysis. Representative quantification of luciferase analysis from three independent experiments was shown. *, $p < 0.05$ t-test vs. the control.

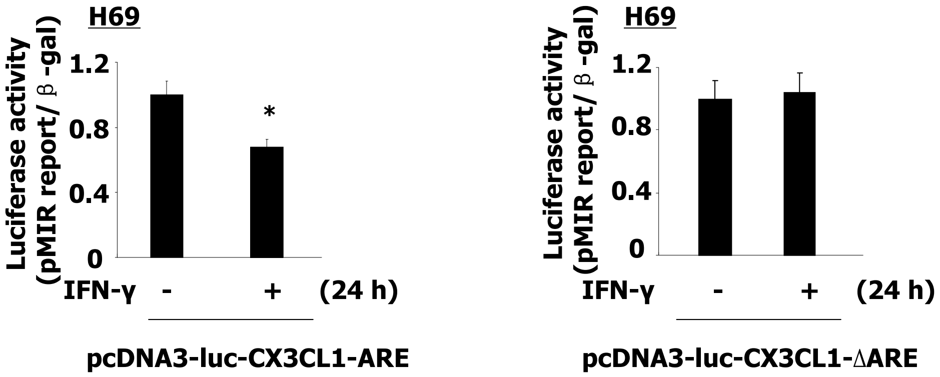

Table S1. Primers used for PCR and sequence used for construct generating.

	Sense primer (5′-3′)	Antisense primer (5′-3′)
**Primers for real-time PCR**		
**Human KSRP**	GCCAAGATGATGCTGGATGA	TTCCTGCAGCTGCTTAATGGT
**Mouse KSRP**	GATGCTGCTACCACCGTGAA	TGCCATCCGGAACCCTATACT
**Human iNOS**	TGCAGACACGTGCGTTACTCC	GGTAGCCAGCATAGCGGATG
**Mouse iNOS**	AGACGGATAGGCAGAGATTGG	ACTGACACTTCGCACAAAGC
**Human COX-2**	CCAGCACTTCACGCATCAGTT	AAAGGCGCAGTTTACGCTGT
**Mouse COX-2**	TGACTGTACCCGGACTGGATT	ATGGGAGTTGGGCAGTCAT
**Human CX3CL1**	CGCAATCATCTTGGAGACGA	GTGCCGCCATTTCGAGTTA
**Mouse CX3CL1**	TTGAGAAGCGGGTGGACAAT	TCCAATGTGGCGGATTCA
**Human GAPDH**	TGCACCACCAACTGCTTAGC	GGCATGGACTGTGGTCATGAG
**Mouse β-ACTIN**	TGGTGGGAATGGGTCAGAA	TCTCCATGTCGTCCCAGTTG
